# PET probe-guided surgery: applications and clinical protocol

**DOI:** 10.1186/1477-7819-5-65

**Published:** 2007-06-07

**Authors:** Seza A Gulec, Erica Hoenie, Richard Hostetter, Douglas Schwartzentruber

**Affiliations:** 1 Center for Cancer Care at Goshen Health System, Goshen, IN, USA

## Abstract

**Introduction:**

Parallel to the advances in diagnostic imaging using positron emission tomography (PET), and availability of new systemic treatment options, the treatment paradigm in oncology has shifted towards more aggressive therapeutic interventions to include cytoreductive techniques and metastasectomies. Intraoperative localization of PET positive recurrent/metastatic lesions can be facilitated using a hand-held PET probe.

**Materials and methods:**

Records of patients who underwent PET probe-guided surgery were reviewed. Surgical indications and operative targets were determined based on diagnostic PET/PET-CT images performed prior to probe-guided surgical planning. PET probe-guided surgery was performed on a separate day using a high-energy gamma probe (PET probe, Care Wise Medical, Morgan Hills CA) 2–6 hours post-injection of 5–15 mCi FDG. Probe count rates, target-to-background ratios, and lesion detection success were analyzed.

**Results:**

Twenty-four patients underwent PET probe-guided surgery; one patient had two PET-probe guided surgeries resulting in a total of 25 cases (5 colorectal cancer cases, 4 thyroid cancer cases, 6 lymphoma cancer cases, and 10 other cancer cases). Surgical indication was diagnostic exploration in 6 cases with lymphoma and 1 case with head and neck cancer (28%). The remaining 18 cases (72%) underwent PET probe-guided surgery with a therapeutic intent in a recurrent or metastatic disease setting. All the lesions identified and targeted on a preoperative FDG-PET scan were detected by the PET probe with satisfactory in-vivo lesion count rates and a TBR of ≥ 1.5. PET probe allowed localization of lesions that were non-palpable and non-obvious at surgical exploration in 8 patients.

**Conclusion:**

The use of the PET probe improves the success of surgical exploration in selected indications. Separate day protocol is clinically feasible allowing for flexible operating room scheduling.

## Background

Gamma probes have been used in medicine and surgery since the mid 1940s. There have been significant technical improvements in probe technology, including a number of clinical applications that have now become standard procedures in contemporary oncologic surgery [1-9]. The introduction of FDG-PET in functional imaging in recent years has markedly improved cancer detection and management. The oncological applications of PET are rapidly expanding with development of new positron-emitting radiopharmaceuticals. Parallel to the advances in PET imaging, and availability of new systemic treatment options, the treatment paradigm in oncology has also shifted towards more aggressive therapeutic interventions including cytoreductive techniques and metastasectomies. This new strategy applies to a number of malignancies including, but not limited to thyroid cancer, neuroendocrine tumors, colorectal cancer (CRC), and melanoma [11-15].

Intraoperative localization of PET-positive recurrent/metastatic lesions can be facilitated using a hand-held PET probe. PET probe essentially is a high energy gamma probe that is designed to process the 511 keV photons of PET tracers. Intraoperative gamma probe performance, as a general rule, is a function of radiopharmaceutical uptake, clearance kinetics, and probe engineering, all ultimately determining the target to background ratio (TBR) and detection threshold. A minimum TBR of 1.5:1 is needed in the operative field for the operating surgeon to be comfortable that the differences between tumor tissue and normal adjacent tissue are real [10]. Due to the high energy photon fluxes, achieving a satisfactory TBR intraoperatively is highly challenging. As such, the clinical use of PET probes has been limited to clinical trial settings, and no standard PET probe-guided surgery protocol has been developed.

PET probe is used routinely in the surgical oncology practice at the Center for Cancer Care at Goshen Health System. This paper discusses the indications and the clinical utility of the PET probe technique, and a PET probe-guided surgery protocol.

## Materials and methods

### Study design and conduct

This study is a retrospective review of 25 cases collected over a period of 18 months. Clinical and operative reports, imaging data and pathology reports were reviewed. Chart review was conducted with the approval of Institutional Review Board and with adherence to HIPAA rules. The objectives of this study were to validate (establish) a clinical protocol for PET probe-guided surgery, and to assess the clinical utility of the technique.

### PET probe-guided surgery protocol

All patients underwent an FDG-PET study either using a stand-alone PET system or a PET-CT scanner under standard clinical protocol. Images were obtained 60–90 minutes after FDG administration. Images were reviewed by a nuclear physician and a surgical oncologist. Standard uptake values (SUV) of the target lesion(s) were measured. The PET probe-guided surgery decision was made after careful review of the imaging information and the clinical indications at a multidisciplinary conference.

The patients received an intravenous injection of 5–15 mCi FDG the morning of planned surgery. Physical activity was kept to a minimum with a quiet time of 60 minutes post-injection. Patients were routinely hydrated with 100–150 ml/hr NS infusion. Surgical exploration was scheduled between 2–6 hours post injection of the radiopharmaceutical. Higher FDG activity was chosen if the surgery was scheduled more than six hours post injection. No glucose containing IV fluids were allowed prior to and during the operation. Relevant images/views were made available for viewing in the operating room.

A high-energy gamma probe with a GSO crystal and 12.5 mm tungsten shielding (Care Wise Medical, Morgan Hills, CA) were used. The Analyzer was set for a photopeak of 511 keV, Window of 20%, and a threshold of 490 keV. Calibration of the system and appropriate settings were verified prior to each operation. The probe with its connecting cord was placed in a plastic sleeve. Surgical exploration commenced with determining the probe survey field. Initial probe survey is performed using the count per second mode. A TBR of 1.5 and above was used for confirmation of the target localization.

## Results

### Patient, disease and lesion characteristics

Records of 24 patients (10 women, 14 men) ages 21–82 years were reviewed. All patients had a whole body FDG-PET scan and a diagnostic CT imaging. A conformational surgical pathology report was available for each case.

Working diagnoses were CRC in 4 patients (5 cases), thyroid cancer in 4, lymphoma in 6, breast cancer in 1, melanoma in 1, adrenocortical cancer in 1, gastrointestinal stromal tumor in 1, gastric cancer in 2, ovarian cancer in 1, head and neck cancer in 2, and lung cancer in 1 patient. PET probe-guided exploration was conducted in the neck in 7 (thyroid cancer: 4, lymphoma: 1, head and neck cancer: 2), axilla in 3 (breast cancer: 1, lymphoma: 2), groin in 3 (lymphoma: 2, melanoma: 1), abdomen in 9 (colorectal cancer: 5, GIST: 1, gastric cancer: 2, ovarian cancer: 1), neck and mediastinum in 1 (thyroid cancer), mediastinum and abdomen in 1 (adrenocortical cancer), and in chest in 1 (lung cancer). Patient and disease characteristics are listed in Table 1.

**Table 1:** Disease, surgical procedure, and clinical outcome report TBR values indicate intraoperative probe measurements

**Case #**	**Diagnosis**	**Operation**	**Probe Utility**	**Time (hrs)**	**SUV**	**TBR**	**Count/Sec**
1	Lymphoma	Excisional Biopsy, Groin	Localized the non-palpable target (A)	6	7.1	2.0	302
2	Recurrent non-Iodine avid Thyroid Cancer	Anterior Neck Dissection	Localized non-palpable metastatic lymph nodes (A)	6	6.4	1.5	166
3	Recurrent non-Iodine avid Thyroid Cancer	Central Neck Dissection	Localized the non-palpable target (A)	6	7.0	1.6	546
4	Adrenocortical Cancer	Sternotomy/Laparatomy Lung/Liver Resection	Localized difficult to access metastatic lymph nodes (A)	6	4.0	1.8	450
5	Ovarian Cancer	Exploratory Laparatomy Metastasectomy	Confirmatory (B)	4	39.8	1.5	297
6	Gastric Cancer	Gastrectomy Extended Node dissection	Localized surgically occult node (A)	4	6.1	1.8	150
7	Colon Cancer	Exploratory Laparatomy Periaortic Dissection	Localized difficult to access metastatic lymph nodes (A)	2	9.1	1.5	1067
8	Lung	Thoracotomy	Confirmatory (B)	4	4.0	1.5	1152
9	Lymphoma Axilla Lymph Node	Excision	Confirmatory (B)	6	4.4	1.8	125
10	Groin Lymphoma	Excision	Confirmatory (B)	4	4.9	2.2	172
11	GIST-Pertonial Implant	Exploratory cytoreduction	Confirmatory (B)	6	9.5	1.9	165
12	Recurrent non-iodine avid Thyroid Cancer	Central Neck Dissection	Localized non-palpable metastatic lymph nodes (A)	6	19.6	2.4	540
13	Lymphoma	Excisional Biopsy, Neck	Localized the non-palpable target (A)	6	7.7	3.8	173
14	Lymphoma	Excisional Biopsy, Groin	Localized the non-palpable target (A)	6	3.6	1.5	749
15	Gastrointestinal Stromal Tumor	Exploratory Laparatomy Liver Resection	Confirmatory (B)	6	4.3	1.5	690
16	Recurrent non-iodine avid Thyroid Cancer	Neck and Mediastinal Node dissection	Localized difficult to access metastatic lymph nodes (A)	6	7.7	1.7	361
17	Lymphoma	Excisional Biopsy, Axilla	Localized the non-palpable target (A)	6	3.3	1.8	828
18	Breast Cancer	Axillary Dissection	Confirmatory (B)	4	3.6	1.8	754
19	Colon Cancer	Liver Resection Exploratory Laparatomy	Confirmatory (B)	6	12.5	1.8	148
20	Branchial cancer	Excision	Localized the non-palpable target (A)	6	9.6	1.5	602
21	Colon Cancer	Laparatomy Celiac Node Dissection	Localized difficult to access metastatic lymph nodes (A)	4	6.1	2.0	127
22	Unknown Primary – Thigh Soft Tissue	Excisional Biopsy	Negative surgical exploration (C)	6	15.8	N/A	N/A
23	Colon Cancer	Liver Resection Exploratory Laparatomy	Confirmatory (B)	6	5.2	3.8	150
24	Colon Cancer	Exploratory Laparatomy Periaortic Dissection	Localized difficult to access metastatic lesions (A)	6	4.5	2.1	894
25	Head and Neck Cancer	Escisional biopsy	Localized the non-palpable target (A)	4	8	2	210

Outcome Legend:

**(A)**: Category-A where the probe's use was instrumental and resulted in direct surgical benefit. **(B)**: Category-B where the probe's use was confirmatory with no direct impact on surgical performance. **(C)**: Category-C where the probe did not locate the image-positive lesion.

### Quantitative measurements and probe performance

PET probe-guided surgical exploration indications were based on the presence of at least one target lesion. All target lesions had an SUV > 3 (Range: 3.2–39.8). PET probe detected all FDG-PET image positive lesions. None of the lesions were immediately apparent at the surgical exploration. The smallest detectable lesion was 0.8 cm. The probe did not identify any lesion(s) that were not seen in preoperative imaging. Surgery was performed at 2 h post-injection in 1 case, at 4 h post-injection in 7, and at 6 h post-injection in 17 cases. No compromise in probe sensitivity was noted up to 6 h post-injection with 15 mCi administered activity. A trend towards an increase in TBR was observed with increasing time interval between the injection and surgical exploration, however, this difference was not statistically significant.

**Figure 1:**
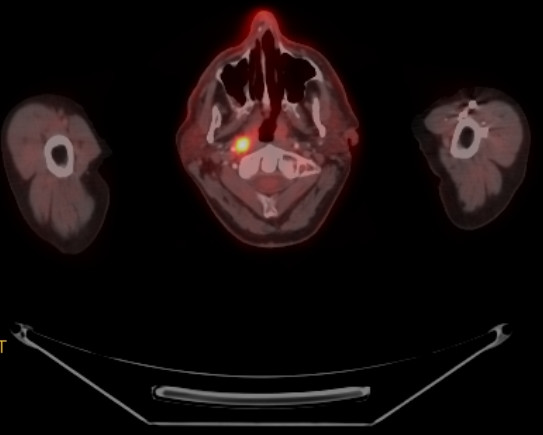
FDG-PET/CT scan of a patient with nasopharyngeal cancer. Transverse slice demonstrating FDG-positive primary site

**Figure 2:**
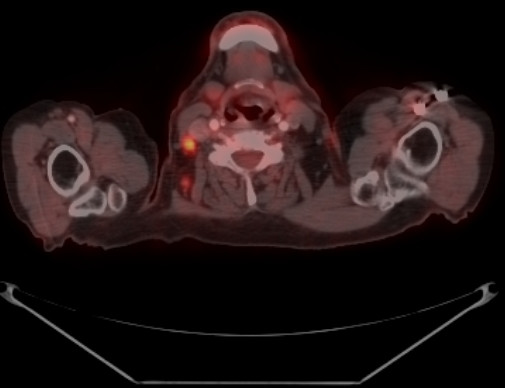
Transverse slice demonstrating FDG-positive lymph node. An US-guided FNA of this node was non-diagnostic.

**Figure 3:**
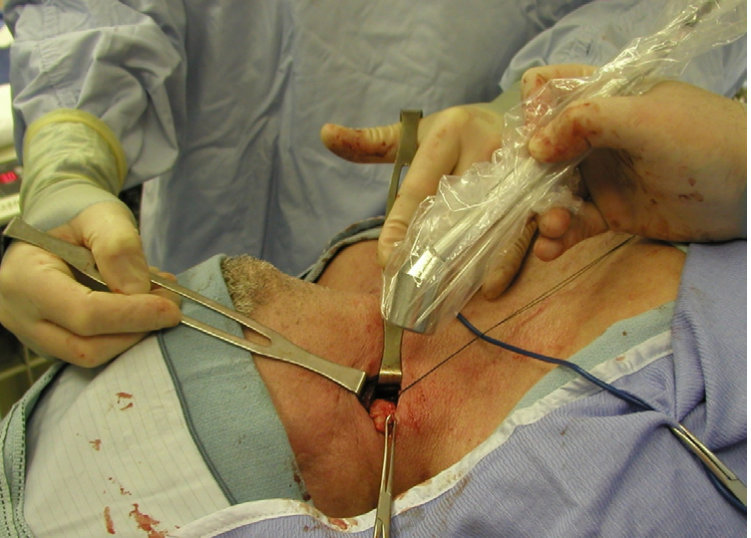
PET-probe guided excision of FDG-positive lymph node in the neck. Final pathology confirmed metastatic squamous cell cancer.

In patients with lymphoma the PET probe was used to locate a non-palpable lymph node during a diagnostic lymphadenectomy. The PET probe successfully located the target lymph nodes (neck, axilla and groin) in all cases. Figures 1, 2, 3 show a case of head and neck cancer where PET probe was used for diagnostic lymphadenectomy in the neck. In patients who underwent a metastasectomy procedure, surgical exploration was clearly facilitated by the use of the probe. It helped lead to the successful accomplishment of the surgical end-point (resection of the image-detected lesion). Probe-guided exploration was most rewarding in secondary explorations where the lesion(s) were obscured by the scar tissue. Manipulation of the probe in the surgical field was easy without any access difficulties. PET probe-guided surgery protocol is detailed in Table 2.

**Table 2:** PET probe-guided surgery protocol for F-18 FDG

**FDG-positive Tumor/Lesion Localization Protocol**	
Radiopharmaceutical	• F-18-FDG
Activity/Administration	• 5–15 mCi/IV injection (Use higher doses if the operation is scheduled more than 4 h post-injection)
Standard Imaging Protocol	• Performed using standard clinical protocol
Timing of Surgical Exploration	• 2–6 h post-injection
Patient Preparation	• NPO × 6 hours
	• Blood glucose control in diabetics (Blood glucose ≤ 140 at the time of FDG injection)
	• 1 hour quiet time
	• Hydration with Normal Saline @ 100–150 cc/h (No glucose containing fluids)
	• Consider β-blockers ± Diazepam for head and neck cases
Gamma Probe	• PET probe (High-energy gamma probe with photopeak detection capability over at 511 keV)
System set-up	• Analyzer Settings: Photopeak: 511 keV, Window: 20%, Threshold: 490 keV (In commercial systems this is obtained by switching the isotope selection to *PET *(FDG) setting)
	• Verify calibration and settings of the system
	• Cover the probe with sterile plastic sleeve
Intra-operative Use	• Point probe tip away from physiologic sites of uptake/accumulation (Foley catheter avoids bladder background)
	• Probe survey at *counts- per-second *mode (Dynamic pitch range feed-back helpful)
	• Hot-spot confirmation with TBR > 1.5 at *10-second count *mode (TBR ratio feed-back helpful)
	• Avoid simultaneous electrocautery use

## Discussion

The success of a PET probe-guided surgery depends on numerous factors including the FDG avidity of the tumor, timing of surgical exploration in reference to injection of FDG, anatomic location of the lesion, its relative proximity to main sites of physiologic uptake/accumulation, and technical properties of the probe. The current study was performed to review the performance of the PET probe in surgical practice, and to validate/establish the optimal surgical protocol. Surgical performance was evaluated based on the probe's the ability to identify lesions seen on diagnostic imaging, and more importantly, actual contribution to the surgical exploration.

All the lesions identified on imaging studies were detected by the PET probe. The size of the lesions varied from 0.8 cm to 4 cm. The PET probe, however, was clearly most useful in detection of surgically occult lesions. Occult lesion was defined as a lesion that was not easily seen or palpated during routine surgical exploration. Some occult lesions included tumor masses measuring 2 cm or above. These were located in regions where the immediate surgical access was difficult due to a barrier of adhesions or scar tissue. A posterior mediastinal exploration and two abdominal re-explorations for recurrent nodal disease involving periportal and celiac regions that were complicated with severe scarring were successfully completed using the PET probe. PET probe effectively directed the surgeon to the lesion(s) which were not found on initial visual and manual exploration. In a patient with metastatic gastric cancer, a N2 node was located using the probe and included in the resection which otherwise could have been retained following the planned gastrectomy. Locating a lesion that is expected to be masked under a scar tissue is probably the strongest indication for PET probe-guided exploration. In a diagnostic setting, we have found PET probe to be very useful in patients with lymphoma who require restaging or regrading of their disease in follow-up. The patients most suitable for this approach are those presenting with non-palpable but FDG-PET positive lymph nodes in axilla, where a surgical dissection is relatively more challenging than that of the groin or neck.

The design of a probe that has the ability to process 511 keV photons, and its intraoperative use against significant background activity is technically challenging. We have previously demonstrated the efficacy and feasibility of PET probe-guided surgical exploration in different clinical settings in a phase II diagnostic study [10]. The technical performance of a probe system is determined by detector sensitivity, spectral resolution, scatter rejection electronics, and shielding [11]. Optimum lesion detectability requires high counting efficiency, an adequate shielding method, and electronics capable of discriminating target signal from radiation noise. Our review of data revealed that there was a trend of TBR improving over time. Improved TBRs (although they were not statistically significant) were observed six hours after FDG administration. There was satisfactory count rate and TBR for surgical detection up to 6 hours post-administration of 15 mCi activity, which makes this technique clinically feasible.

Detector material for the probe plays an important role in the sensitivity of the system. A GSO crystal is known to have a better stopping power than the NaI or CsI crystals and much better efficiency than the semiconductors used in medium energy gamma probes [16]. The PET probe also has to have heavier shielding. The profile, size and the weight of the probe and the ergonomics of the probe have to be non-restrictive to surgical exploration. The analyzer of the probe system allows exclusion of scattered radiation which determines the specificity of the system. Therefore, analyzer electronics are equally (if not more) important in the overall performance of the probe system. Poor electronic suppression of scattered photons seriously degrades the TBR. This can cause small target lesions to be missed in the background. Effective electronic suppression of scattered photons requires a precisely placed photon energy acceptance window set atop a scatter rejecting threshold.

FDG avidity is determined by glycolytic activity of the tumor and the viable tumor concentration in a given lesion. This feature contributes to the success of probe detection; however, it has nothing to do with the specificity. Individual cancer types may also show significant variability in terms of FDG avidity. Even in the same patient, different lesions may have different degrees of FDG uptake. The probe detects any FDG avid lesion whether it is malignant or inflammatory.

FDG metabolism and clearance occurs at a much faster rate in normal tissues than tumor tissue, and thus TBR improves with time resulting in better lesion detection when imaging is delayed [17]. We have observed that tumor-to-non-tumor and tumor-to-organ rations were higher for the delayed images than for the 1.5-h routine images, and lesion detectability was improved in nodal and hepatic metastases. Our current study indicated that longer intervals accentuated the TBR, and resulted better lesion detection. The background radiation tends to decrease while the tumor uptake is retained. The in-situ TBR is also strongly affected by the areas of physiologic uptake or accumulation. The brain uptake in the head and neck region, cardiac uptake in the chest, kidney uptake and the accumulation inside the bladder in the abdomen and pelvis affect the in-situ TBR. Areas of physiologic uptake show attenuation over time, and use of an intraoperative bladder catheterization minimizes interference from bladder accumulation of FDG.

The role of surgery in recurrent/metastatic cancer is being redefined. With the advance of tools or early diagnosis, and the improvements in systemic therapy, metastasectomy and cytoreductive surgical techniques in selected cases are considered viable management options. The clinical indications for metastasectomy and/or cytoreductive resections are beyond the scope of this discussion. Parallel to the development of techniques to identify favorable tumor biology, and having the ability to select patients who are expected to have a more protracted disease course (such as in differentiated thyroid cancer and neuroendocrine tumors) or tumors which are responsive to systemic therapies (such as colorectal cancer and breast cancer) more patients will be considered for metastasectomy. PET probe, with further refinements in the design and technical performance, might prove to be a very useful tool in surgical management of recurrent/metastatic disease. This study demonstrates the technical ability and feasibility of FDG-positive lesion detection using a PET probe.

Currently, with all recognized limitations, FDG imaging represents a new standard for functional/biologic imaging in oncology. Many more new PET radiopharmaceuticals are being developed for distinct tumor types and tumor phenotypes [18,19]. Peptide-based agents, kinetically and diagnostically, possess the most favorable radiochemistry. Cu-64 and Ga-68 labeled octreotide are being increasingly utilized in detection of occult neuroendocrine tumors [20]. All PET radiopharmaceuticals have unique biodistribution characteristics defining different in-situ TBR profiles, some clearly yielding much better TBR than that of FDG in a given lesion.

## Conclusion

The clinical indications of the PET probe will individually be determined based on the tumor and patient characteristics as well as the biodistribution patterns of the selected PET radiopharmaceuticals. In carefully selected indications, PET probe can be considered as a useful adjunct in surgical practice.

## Competing interests

Research support from Care Wise Medical, Morgan Hills, CA

## Authors' contributions

**SG **– Design, Acquisition, analysis and interpretation of data, drafting manuscript, critical revision

**EH **– Drafting and editing of manuscript, data analysis

**RH **– Acquisition of data, critical revision

**DS **– Critical revision

All authors read and approved the final manuscript
